# Double Trouble: A Unique Case of Hypercalcemia Caused by Two Underlying Etiologies

**DOI:** 10.7759/cureus.21084

**Published:** 2022-01-10

**Authors:** Eloy E Ordaya, Jose Arriola-Montenegro, Liliana Arriola-Montenegro, Mel L Anderson

**Affiliations:** 1 Infectious Diseases, Mayo Clinic, Rochester, USA; 2 Internal Medicine, University of Minnesota, Minneapolis, USA; 3 Internal Medicine, Universidad Peruana de Ciencias Aplicadas, Lima, PER; 4 Internal Medicine, Rocky Mountain Regional VA Medical Center, University of Colorado, Aurora, USA

**Keywords:** granulomatous infection, occult malignancy, hypercalcemia, diffuse large b lymphoma, colonic actinomycosis

## Abstract

Hypercalcemia has a variety of causes, with primary hyperparathyroidism and malignancies being the most frequently reported. We present the case of a patient presenting with chronic abdominal pain, constipation, and weight loss who was found to have hypercalcemia. The patient was initially diagnosed with colonic actinomycosis, but further investigations revealed an intra-abdominal diffuse large B-cell lymphoma (DLBCL). We suspect that the leading cause of hypercalcemia was the DLBCL, likely exacerbated by actinomycosis. Actinomycosis and DLBCL can have a similar presentation, so misdiagnosis or coexistence of both conditions should be suspected when a lack of response to one specific therapy is observed.

## Introduction

Hypercalcemia is a common medical problem that frequently presents with abdominal pain, constipation, and altered mental status. There are many etiologies of hypercalcemia but the most common are primary hyperparathyroidism and malignancies [1,2]. Malignancies can cause hypercalcemia through various mechanisms, including the production of PTH-related peptide (PTHrP), osteolytic metastasis, and increased production of 1,25-dihydroxyvitamin D (1,25-(OH)2D) [1,3]. Granulomatous infections, such as actinomycosis, can increase the production of 1,25-(OH)2D, producing hypercalcemia by a similar mechanism as seen in certain types of lymphomas [4,5]. Herein, we present the case of a patient with symptomatic hypercalcemia caused by a diffuse large B-cell lymphoma (DLBCL), probably exacerbated by concurrent actinomycosis.

## Case presentation

A 77-year-old man with diabetes mellitus, hypertension, and chronic kidney disease presented at the clinic with abdominal pain, constipation, and a ten-pound weight loss during the previous eight months. He denied fever, melena, or hematochezia. On presentation, he was afebrile and normotensive, with laboratory testing remarkable for calcium of 11.5 mg/dL (normal range: 8.6 - 10.3 mg/dL). One week later, the repeated calcium test was still elevated and complementary blood testing was done. The results are shown in Table 1.

**Table 1:** Relevant hypercalcemia work-up testing before hospitalization 1,25-(OH)2D: 1,25-dihydroxyvitamin D; 25-(OH)D: 25-hydroxyvitamin D; PTH: parathyroid hormone; PTHrp: parathyroid hormone-related protein.

Laboratory test	Value	Normal range
Calcium (mg/dL)	11.8	8.6 - 10.3
Albumin (g/dL)	3.7	3.5 – 5
Intact PTH (pg/mL)	< 6.3	11.1 – 79.5
PTHrp (pg/mL)	17	14 – 27
1,25-(OH)2D (pg/mL)	91	20 - 60
25-(OH)D (pg/mL)	25	25 – 80

**Figure 1:**
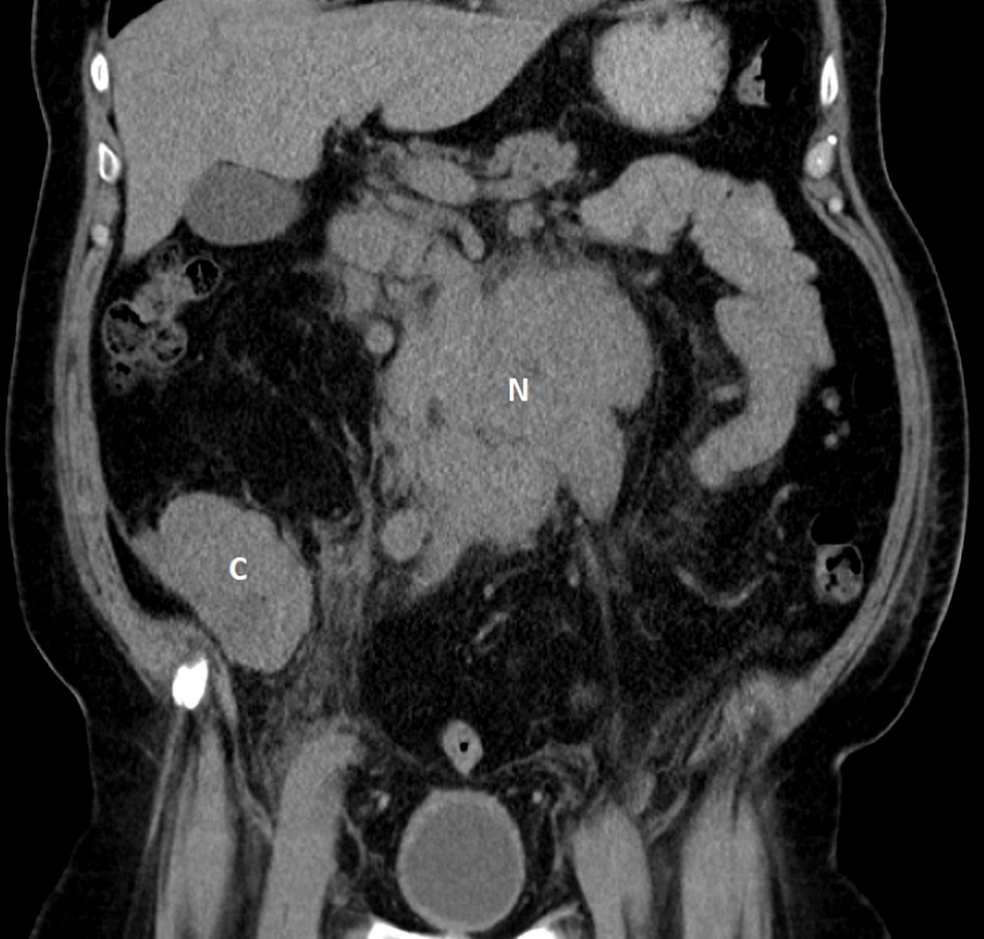
Computed tomography of the abdomen (coronal plane) showing a severe cecal wall thickening (C) and a conglomerate lymph nodal mass (N)

The patient was initially treated with hydration and by eliminating factors that can exacerbate hypercalcemia, including avoidance of prolonged bed rest and calcium supplementation. Despite this management, a repeat calcium test showed 13.5 mg/dL. Due to high suspicion for malignancy as a cause of hypercalcemia, he underwent computed tomography (CT) of the abdomen that showed cecal thickening and enlarged pelvic, mesenteric, and retroperitoneal lymph nodes (Figure 1).

**Figure 2:**
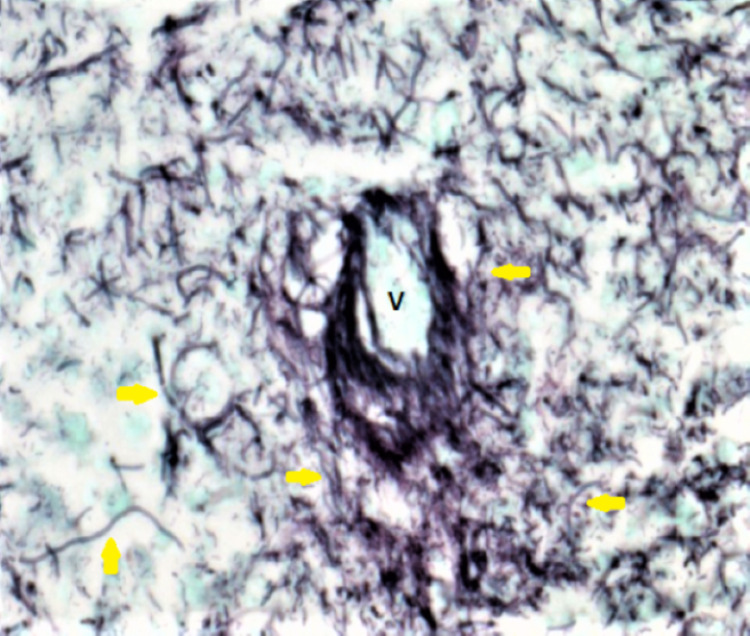
Cecal mass biopsy: Gomori methenamine silver (GMS) stain showing filamentous organisms compatible with Actinomyces species (yellow arrows) around a dead vessel (V) (200x magnification)

The patient underwent a colonoscopy that revealed an infiltrative, exophytic cecal mass with histopathological examination reporting an ulcerated mucosa with abundant *Actinomyces* organisms without malignant cells (Figure 2). Therefore, the patient was admitted to the hospital for further management.

On admission, his physical exam was remarkable for confusion and tender, palpable masses in the mid-abdomen and right lower quadrant. Laboratory testing is presented in Table 2.

**Table 2:** Laboratory blood tests on hospital admission ALP: alkaline phosphatase; ALT: alanine transaminase; AST: aspartate transaminase; CRP: C-reactive protein; ESR: erythrocyte sedimentation rate; MCV: mean corpuscular volume; WBC: white blood cells.

Laboratory test	Value	Normal range
WBC (10^3^/uL) count	14.5	5 – 10
Hemoglobin (g/dL)	12	13.5 – 16
MCV (fL)	70	80 – 100
Platelets (10^3^/uL)	294	150 – 450
Glucose (mg/dL)	116	70 – 100
Creatinine (mg/dL)	2.3	0.7 – 1.2
Urea (mg/dL)	39	7 – 30
AST (IU/L)	31	8 – 48
ALT (IU/L)	18	7 – 55
ALP (IU/L)	80	40 – 129
Total protein (g/dL)	5.5	6.3 – 7.9
Albumin (g/dL)	2.8	3.5 – 5
CRP (mg/L)	92	0 – 3
ESR (mm/hr)	39	0 – 22
Calcium (mg/dL)	11.8	8.6 - 10.3
Ionized calcium (mg/dL)	6.4	4.4 – 5.2

**Figure 3:**
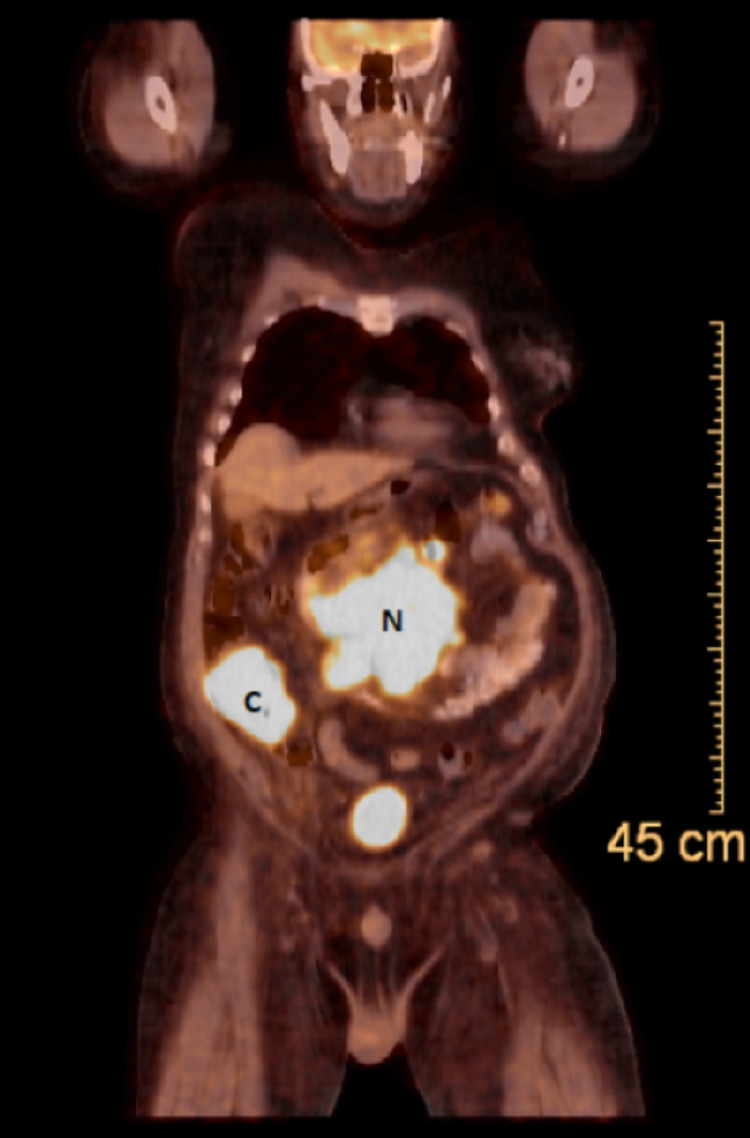
Positron emission tomography showing intense FDG uptake of the cecum (C) and retroperitoneal lymph nodal mass (N)

Initial management included intravenous hydration, calcitonin, and pamidronate for hypercalcemia, and penicillin G for actinomycosis treatment. During the following days, his confusion, leukocytosis, and hypercalcemia resolved, and his creatinine improved. Given his clinical presentation and imaging findings concerning for a concurrent malignant etiology, a positron emission tomography (PET) was done and showed fluorodeoxyglucose (FDG) avid of the cecum and retroperitoneal lymph nodal mass (Figure 3).

**Figure 4:**
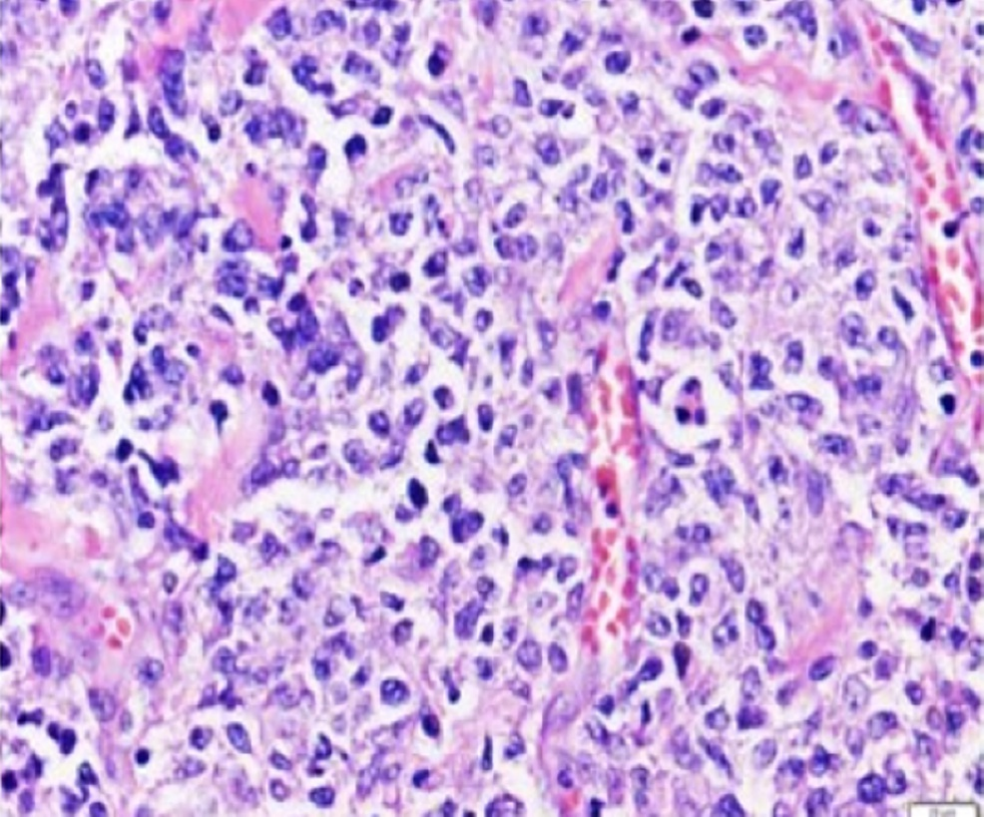
Cecal mass biopsy showing large and pleomorphic lymphoid cells (200x magnification)

Fine needle aspiration of the lymph nodal mass revealed a diffuse large B-cell lymphoma (DLBCL). The patient received treatment with prednisone and rasburicase as pretreatment for DLBCL, but days later the patient developed melena with a hemoglobin drop to 6.7 mg/dL. Repeat colonoscopy showed an ulcerated, obstructing cecal mass. The patient underwent an ileocecal resection and ileostomy, with biopsy of the resected cecal mass reporting DLBCL without evidence of *Actinomyces* organisms (Figure 4).

Therefore, penicillin G was discontinued after completing two weeks of therapy, and chemotherapy with rituximab and vincristine was started. The patient was discharged and completed six cycles of chemotherapy, obtaining remission.

## Discussion

Understanding the mechanisms of hypercalcemia may help to define the primary underlying disorder behind the elevation of calcium. Malignancies can produce hypercalcemia by various mechanisms. Different types of cancer, including squamous cell tumors and hematologic malignancies, can produce PTHrP that causes hypercalcemia without malignant bone involvement [1]. Malignancies arising from or that metastasize to the bone overproduce growth factors and cytokines that enhance osteolysis, releasing calcium from the bone [1]. Other malignancies overexpress 1,25(OH)2D-synthesizing enzyme CYP27b1, increasing the conversion of 25-hydroxyvitamin D to 1,25(OH)2D. This mechanism is seen in 5-15% of lymphomas and produces hypercalcemia by increasing the intestinal and bone reabsorption of calcium [1,3]. Granulomatous diseases increase the production of 1,25(OH)2D, but unlike lymphomas, it is produced by activated mononuclear cells in the lungs and lymph nodes [3]. These two latter mechanisms could have contributed to the hypercalcemia seen in this patient.

The initial treatment of hypercalcemia includes avoidance of precipitation factors, hydration, and subcutaneous or intramuscular calcitonin. Calcitonin lowers calcium levels by inhibiting bone resorption and is used irrespectively of the etiology of hypercalcemia due to its rapid onset of action [2]. Intravenous bisphosphonates are particularly useful for malignancy-associated hypercalcemia. They act by inhibiting the osteoclast-mediated bone resorption, inducing osteoclast apoptosis, and decreasing osteoblast apoptosis [6,7]. Despite their proven efficacy, it may take several days to see their full effect; thus, calcitonin should be used initially [2]. Glucocorticoids are also part of the management of hypercalcemia, especially when it is secondary to lymphomas and granulomatous diseases. They decrease the intestinal absorption of calcium by reducing the synthesis of 1,25(OH)2D and increasing urinary calcium excretion [8].

Actinomycosis was considered the initial cause of hypercalcemia in this patient, but given his clinical features and imaging findings, a malignancy was suspected. *Actinomyces* spp. are filamentous gram-positive bacilli that are normal inhabitants of the mouth and intestinal tract and can cause cervicofacial, and less commonly, thoracic, abdominal, or pelvic infections (particularly in patients with intrauterine devices) [9]. Abdominal actinomycosis is generally caused by an injury in the gastrointestinal mucosa through abdominal surgery or external injury that predisposes the invasion of the bacteria. However, it may also be precipitated by prolonged use of glucocorticoids or chemotherapy [10]. This infection can resemble other chronic diseases or present as acute abdominal pain and often goes undiagnosed until surgery or obtaining biopsy results [11,12]. Actinomycosis can also mask malignancies, such as lymphomas, which may cause the initial mucosal injury producing the substrate for the microorganism to reproduce and invade [13,14]. In our patient, we think that a localized cecal actinomycosis hid the DLBCL. Treating this infection and performing a histopathological examination of the resected cecal mass led to the final diagnosis. There are other reports of gastrointestinal lymphomas initially mistaken for actinomycosis [14,15].

In this case, we suspect that the intra-abdominal DLBCL was the most likely cause of hypercalcemia, worsened by concurrent localized cecal actinomycosis infection. Given the similar clinical presentation of both conditions, we should emphasize the importance of performing a thorough diagnostic evaluation when DLBCL or actinomycosis are diagnosed to rule out their coexistence.

## Conclusions

We present a unique case of lymphoma causing hypercalcemia, likely worsened by *Actinomyces* infection. Determining the etiology of hypercalcemia can be challenging for clinicians, but it is essential to identify the underlying disorder(s) as that will guide the management. The treatment of hypercalcemia of malignancy is based on hydration, calcitonin, bisphosphonates, glucocorticoids, and treatment for the underlying disease.
